# Supplementary material for: Re-Sensitization of Resistant Ovarian Cancer SKOV3/CDDP Cells to Cisplatin by Curcumin Pre-Treatment
Source: Int J Mol Sci. 2025 Jan 18;26(2):799. doi: 10.3390/ijms26020799 (PMC11765683; doi:10.3390/ijms26020799)
Supplement: Supplementary file 1 [file ijms-26-00799-s001.zip › ijms-3370320-supplementary.pdf]

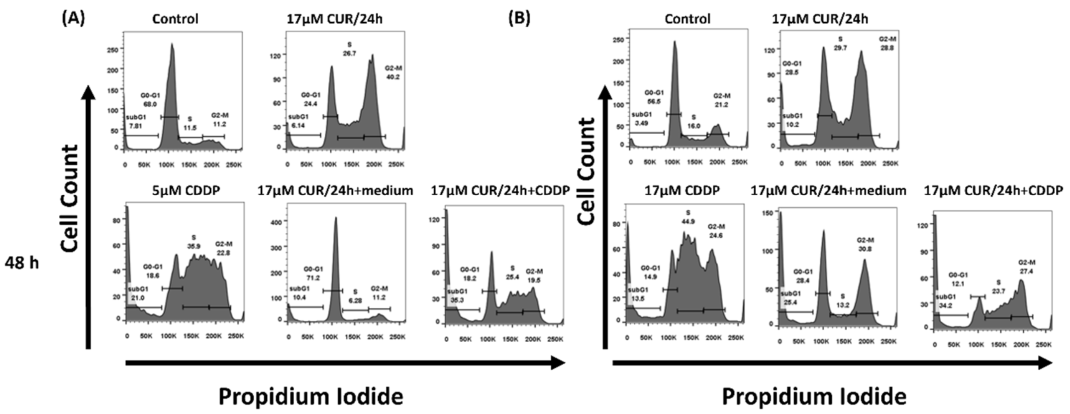

Figure S1. Cell cycle analysis by flow cytometry in ovarian (**A**) SKOV-3 and (**B**) SKOV-3/CDDP cancer cell lines treated with either (1/2 IC_{50}, 5 μM or 17 μM for SKOV-3 and SKOV-3/CDDP cell lines, respectively) CDDP for 48 h alone or (17 μM) CUR for 24 h then the medium was replaced with fresh culture medium for 48 h alone or CUR pre-treatment by treatment firstly with (17 μM) CUR for 24 h followed by treatment with CDDP for another 48 h (5 μM or 17 μM for SKOV-3 and SKOV-3/CDDP cell lines, respectively).

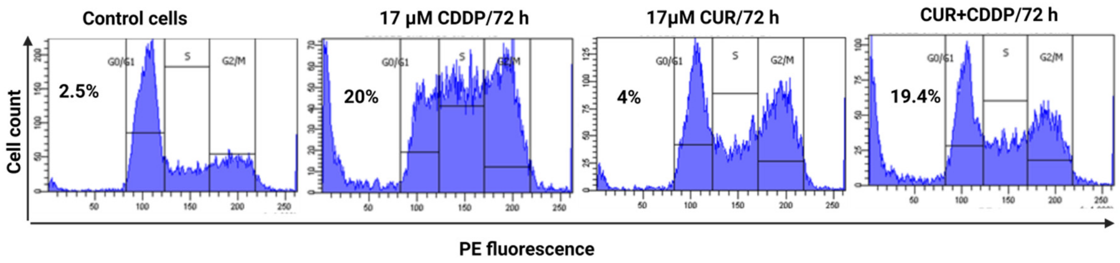

Figure S2. Cell-cycle analysis by flow cytometry in ovarian SKOV-3/CDDP cancer cell lines treated with the combination of CUR+CDDP for 72 h.

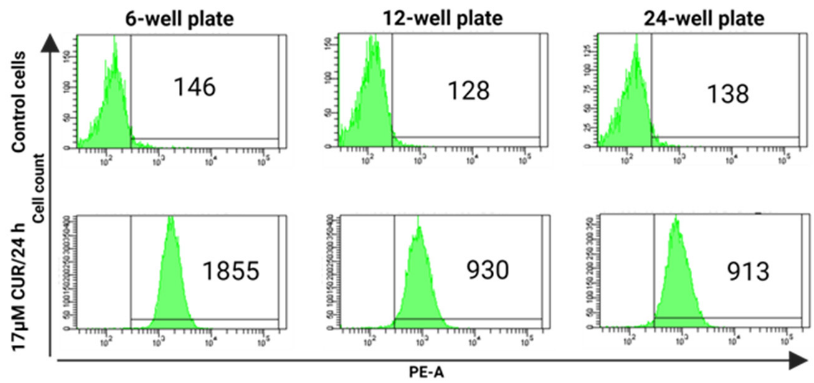

Figure S3. Intracellular CUR accumulation at different cell densities was assessed by flow cytometry.
